# Multi-level barriers and facilitators to buprenorphine use in Ontario, Canada: a qualitative study using the theoretical domains framework

**DOI:** 10.1186/s13722-025-00610-w

**Published:** 2025-10-21

**Authors:** Pamela Leece, Triti Khorasheh, Suzanne Zerger, Kim Corace, Lara Nixon, Carol Strike, Ahmed M. Bayomi, Elisabeth Marks, Melissa Holowaty, Frank Crichlow, Sheena Taha, Sharon E. Straus

**Affiliations:** 1 Public Health Ontario, Toronto, ON Canada; 2 Dalla Lana School of Public Health, University of Toronto, Toronto, ON Canada; 3 Department of Family and Community Medicine, University of Toronto, Toronto, ON Canada; 4 Department of Psychiatry, University of Ottawa, Ottawa, ON Canada; 5 Canadian Centre on Substance Use and Addiction, Ottawa, ON Canada; 6 Cumming School of Medicine, University of Calgary, Calgary, AB Canada; 7 MAP Centre for Urban Health Solutions, La Ka Shing Knowledge Institute, Unity Health Toronto, Toronto, ON Canada; 8 Department of Medicine, University of Toronto, Toronto, ON Canada; 9 Department of Family Medicine, Queen’s University, Kingston, ON Canada; 10 Canadian Association of People Who Use Drugs (CAPUD), Ottawa, ON Canada; 11 Knowledge Translation Program, La Ki Shing Knowledge Institute, Unity Health Toronto, Toronto, ON Canada; 12 Public Health Ontario, 661 University Avenue, Suite 1701, Toronto, ON M5G 1M1 Canada

**Keywords:** Buprenorphine, Opioid agonist treatment, Opioid use disorder, Implementation

## Abstract

**Background:**

Few studies have systematically examined the barriers and facilitators to buprenorphine uptake, despite increasing opioid-related harms and guideline recommendations for use. The aim of the study was to use behaviour change frameworks to investigate barriers and facilitators to buprenorphine access and use from diverse perspectives in Ontario, Canada.

**Methods:**

We conducted semi-structured face-to-face or telephone interviews with Ontarians including: people with living/lived expertise of opioid use (including family members), healthcare professionals, and organizational and system-level representatives. We used purposive sampling via existing professional networks to recruit participants with diverse experiences. The Theoretical Domains Framework (TDF) guided the data collection tool and analysis. Interviews were recorded, transcribed, coded, and underwent thematic analysis involving three study team members.

**Results:**

We interviewed 28 participants between September 2019 and January 2020. Three predominant TDF domains were identified across all 4 levels: (1) environmental context/resources; (2) beliefs about consequences; (3) social influences. Key cross-cutting themes included access to comprehensive care, medication and treatment characteristics, confidence and experiences with buprenorphine, as well as supportive relationships and stigma/discrimination.

**Conclusions:**

Multi-level barriers to optimal buprenorphine implementation continue in the face of the drug toxicity crisis. To counter the identified barriers and enhance facilitators, there is need for mentorship models of support for prescribing, flexibility in buprenorphine treatment requirements, better recognition of mental health and the social determinants of health in buprenorphine treatment, and comprehensive and integrated systems of care.

**Supplementary Information:**

The online version contains supplementary material available at 10.1186/s13722-025-00610-w.

## Introduction

Fatal and non-fatal opioid toxicity continues to impact people, families, and communities across Canada, with 7,525 apparent opioid-related deaths in 2022 [1]. Substance use treatment is a core health service within the national framework for responding to opioid-related morbidity and mortality [2]. Opioid agonist treatment (OAT) uses pharmacotherapy, such as buprenorphine or methadone, for the long-term treatment of opioid use disorder [3]. Systematic reviews with meta-analyses suggest that both medications are effective in reducing opioid-related morbidity and mortality; however, the superior safety profile of buprenorphine (commonly referred to by the trade name Suboxone) has led to national guidelines recommending it as the preferred first-line therapy over methadone for most population groups [3, 4]. 

Data from Ontario, Canada shows buprenorphine prescribing increased in the past five years [5], yet there remains a gap in access and unmet treatment need for this population [6]. Multi-level barriers to buprenorphine treatment have been documented in the American and Canadian literature. At the patient level, reported barriers to buprenorphine treatment have stemmed from dissatisfaction and negative experiences with the medication and associated side effects [7–9], previous discriminatory interactions with healthcare professionals [7], access issues [8–11], and attitudes towards their treatment (e.g., inflexibility of the program, frustration with delayed tapering processes) [7–9, 12]. Throughout this paper, we use the term “people with living/lived expertise” to refer to the patient-level insights based on feedback from community partners and recent work published by the Canadian Association of People who Use Drugs [13]. Our work follows the principle of “nothing about us without us,” recognizing the public health, ethical, and human rights foundations for involving people who use drugs in decisions that affect their lives [14]. 

Professional barriers in implementing buprenorphine include lack of interest, lack of training, stigma towards the population [15], inadequate resources, and program requirements [16, 17]. Limited access to buprenorphine prescribers is a commonly reported system-level barrier in the United States (U.S.), including challenges related to rural location, cost, and health insurance coverage [18–20]. 

A theory-informed qualitative study of the barriers and facilitators at all levels of the health system can inform population-level changes in the use of buprenorphine as a strategy to reduce mortality within the current drug toxicity crisis. To our knowledge, such an analysis has not been published. An integrated study across levels can point to domains that are common across levels, improving efficiency and focus on interventions that have the most potential for impact. Further, the use of established theoretical frameworks in implementation science can inform the selection of specific behaviour change techniques with evidence of effectiveness in addressing particular domains of behaviour [21, 22]. This approach can align various areas of evidence and action, particularly for complex problems such as the drug toxicity crisis and the need for a strategic health system response. The aim of this study was to use the Theoretical Domains Framework (TDF) to investigate barriers and facilitators to buprenorphine access and use at the patient, healthcare professional, organization, and system levels in Ontario. Using a theory-informed approach, this study aims to provide a strong theoretical basis to guide the development of future strategies to improve buprenorphine implementation.

## Methods

### Study design

This study is part of a larger project to evaluate the implementation of buprenorphine in Ontario and identify strategies to facilitate change in use and access of buprenorphine [23]. The project also includes a scoping review of the literature and a stakeholder workshop to recommend priority actions based on our findings. While the scoping review was initiated earlier, it was completed concurrently with the qualitative study. The stakeholder workshop took place following the completion of both components. In Ontario, physician and hospital services for opioid use disorder are publicly funded, the majority of prescriptions for buprenorphine are written by a small number of high-volume prescribers, and patients receiving social income support have drug coverage for buprenorphine under the Ontario Drug Benefit. We conducted a qualitative study using semi-structured interviews with Ontarians representing four perspectives on buprenorphine access and use: patients/family members (e.g., people with living/lived expertise of opioid use); healthcare professionals (e.g., physician, nurse, pharmacist); organizational leaders (e.g., medical director, executive director); and system leaders (e.g., representatives involved with guidelines, regulations or education across organizations).

We used the TDF to design our data collection tools and map the barriers and facilitators described (Additional File 1). The TDF incorporates 14 constructs from a range of organizational and individual behaviour change theories to understand behaviour related to the implementation of evidence-based recommendations [21]. The TDF can be seen as an elaboration of another framework, the capacity, opportunity, motivation model of behaviour (COM-B), that forms the hub of the Behaviour Change Wheel (BCW) [22]. Using the TDF helps to: (1) classify barriers and facilitators related to a behaviour; and (2) link behaviours to the COM-B hub of the BCW, which can be used for designing strategies that target mechanisms of behaviour change [22]. 

As part of an integrated knowledge translation approach to ensure relevance, feasibility and acceptability, we established an Advisory Committee [24]. The study team and Advisory Committee were made up of researchers and representatives with expertise at each level including, people with living/lived expertise of substance use, addiction medicine physicians, and healthcare leaders. Both groups were actively engaged via regular meetings throughout all stages of the study. The study protocol (2019 − 031.01) was approved by the Ethics Review Board at Public Health Ontario on August 14, 2019. This study used the Standards for Reporting Qualitative Research (Additional File 2) [25]. 

### Participant selection and recruitment

We recruited participants through the professional networks of our study team and Advisory Committee. We used purposive sampling to identify participants based on a matrix developed through team consensus, which helped ensure a diversity of perspectives were included (Table 1). We used this matrix to guide recruitment across broad perspectives rather than outline specific inclusion criteria to select participants. For example, the matrix for healthcare professionals included physicians, pharmacists, and nurse practitioners, in practice for short (< 5 years) and long (> 10 years) durations, working in rural or urban environments, and with a focused or general practice in a hospital, residential, or community setting. Additional participants were identified through snowball sampling.Due to resource limitations, we aimed to conduct at least 20 interviews with five participants covering each of the four levels. The team subsequently increased the number of interviews for lived/living expertise to prioritize the perspectives of people served by buprenorphine treatment and recognize the wide range of diverse experiences among patients in the province. There were a few matrix elements that were not fully achieved, including variation in participant location.

**Table 1:** Matrix for participant recruitment

Perspective	Matrix elements
(1) Patients/family members (e.g., people with living/lived expertise of opioid use)	• Route of opioid use (injection vs. non-injection) • Buprenorphine experience (yes/no) • Family member • Harm reduction worker with lived expertise • Other: gender, age, race, ethnicity
(2) Healthcare professional (e.g., physician, nurse, pharmacist)	• Profession (physician, nurse, pharmacist) • Years in practice • Type of practice • Location (urban, rural) • Practice setting*
(3) Organization leaders (e.g., medical director, executive director)	• Practice setting* • Location (urban, rural) • Current services • Current team-based substance use approach • Buprenorphine provision (full, limited, none)
(4) System leaders (e.g., representatives involved with guidelines, regulations or education across organizations)	• Level (provincial, regional, local) • Program area (pharmacy, nursing, healthcare, corrections, residential/withdrawal management) • Association or regulatory body • Education or supports • Guidance, standards or quality improvement

* Hospital, primary care teams, correctional facilities, residential/withdrawal management

Participants were emailed an interview request with a copy of the consent form and interview guide. Those who expressed interest were asked to provide a convenient date and time, and, when applicable, their preference for in-person or telephone interview. Advisory Committee members with experience in community outreach helped guide the recruitment of participants for the patient/family member perspective through their networks including program staff and posters at two harm reduction programs in Downtown Toronto.

### Data collection

The TDF informed the development of the interview guide [21, 22]. Prior to data collection, the interview guide was piloted with members of our Advisory Committee representing the four levels to assess its comprehensiveness and clarity. Two study members (PL, SZ) each conducted individual semi-structured interviews lasting 30 min to 1-hour via telephone or in-person, including interviews with people with living/lived expertise of opioid use that took place in private rooms at health and social service settings. The interviewers each had relevant research expertise and no prior relationship with participants that would introduce undue influence, although prior professional connections may have occurred. Participants were able to have a personal or professional support person present as needed. All participants provided verbal consent, which interviewers documented in the study records, and received an honorarium.

### Data analysis

Interviews were audio-recorded and transcribed verbatim by a third-party transcription service. The study team ensured all transcripts were de-identified, removing any potentially identifying information (e.g., clinic name or location). Each participant was offered the opportunity to review their own transcript for accuracy and clarity. Three study members (PL, SZ, TK) independently reviewed a set of five transcripts to code excerpts representing a barrier or facilitator into the 14 TDF domains and take notes on preliminary codes. The three members then met to discuss and reach consensus on the coding into the TDF domains, and through discussion developed the coding framework and emerging themes. This process allowed for study team members to clarify and reach a mutual understanding of the TDF domains. While barriers and facilitators were originally coded separately, through discussions, the team agreed to pool barriers and facilitators together, as the same concepts were presented in both positive or negative terms (e.g., experience of stigma as a barrier and absence of stigma as a facilitator). Once consensus was reached, one member (SZ) applied the final coding framework to the rest of the transcripts in NVivo 10 software, using the 14 TDF domains as primary nodes to categorize relevant themes. The remaining two study members (PL, TK) independently reviewed the codes applied to the transcripts to ensure consistency in interpretation and application of the coding framework. After coding, one member (SZ) thematically analyzed the barriers and facilitators for sub-themes within the TDF domains, prepared memos, and calculated the frequency of codes applied to each domain by perspective. Study team and Advisory Committee members reviewed the list of barriers and facilitators and discussed interpretations of themes. We favoured the use of the TDF over the COM-B in our analysis and write-up as it provided more detailed subcategories of the components of behaviour.

## Results

We conducted 28 interviews between September 2019 and January 2020, meeting our planned sample distribution, of whom 11 (39%) self-identified as persons with living/lived expertise; 8 were recruited from Toronto and others from Southern Ontario. Healthcare professionals, organizational, and system leader perspectives were each represented by 5 to 6 people. Respondents predominantly resided in Central Ontario (21; 75%), but were dispersed throughout rural and urban settings (Table 2).

**Table 2:** Participant characteristics

Characteristic	Number (%) of participants *n* = 28
**Geographic region within Ontario**	
North	2 (7%)
East	3 (11%)
West	2 (7%)
Central	21 (75%)
**Perspective**	
(1) People with living/lived expertise of opioid use	11 (39%)
(2) Healthcare professional (e.g., physician, nurse, pharmacist)	6 (21%)
(3) Organizational leader (e.g., medical director, executive director)	5 (18%)
(4) System leader (e.g., involved with guidelines, regulations or education)	6 (21%)

We identified three predominant TDF domains of barriers and facilitators to buprenorphine access and use and themes within each domain that cut across the four perspectives. The TDF domains included: (1) environmental context/resources (comprehensive interdisciplinary care and continuity and accessing healthcare professionals and clinics) (34%); (2) beliefs about consequences (buprenorphine characteristics, treatment characteristics, and confidence and experience) (27%); and (3) social influences (stigma and discrimination, relationships and supports) (11%) (Table 3). We coded fewer than one-tenth of the barriers and facilitators to the other TDF domains. Similar to other studies applying the TDF, we used frequency counts of codes using the TDF to identify and account for the range of barriers and facilitators across our qualitative dataset. However, we focus the subsequent presentation of results on the cross-cutting themes across perspectives rather than the framework. We believe this provides a more rich understanding of barriers and facilitators, action to create larger impact, and overall more feasible than detailed discussion of 14 domains by 4 levels (56 areas).

**Table 3:** Frequency of TDF domains

TDF domain	Percentage of codes
Environmental Context/Resources	34%
Beliefs about Consequences	27%
Social Influences	11%
Professional/Social Role and Identity	4%
Reinforcements	4%
Emotion	4%
Knowledge	3%
Behavioural Regulation	3%
Beliefs about Capabilities	2%
Optimism	2%
Intentions	2%
Goals	2%
Skills	1%
Memory, Attention, and Decision Processes	1%

Table 4 includes the variations by perspective (Additional File 3).

**Table 4:** Top TDF domains by perspective

Perspective	Beliefs about consequences (% of codes)	Environmental context/resources (% of codes)	Social influences (% of codes)	Top 3 TDF domains combined (% of codes)
People with living/lived expertise of opioid use	51%	21%	15%	87%
Healthcare Professional (e.g., physician, nurse, pharmacist)	19%	36%	11%	66%
Organizational leader (e.g., medical director, executive director)	14%	43%	8%	65%
System leader (e.g., involved with guidelines, regulations or education)	16%	40%	8%	64%

The cross-cutting themes and supporting quotes are described below (Additional File 4, Table 5).

**Table 5:** Cross-cutting themes

TDF domain	Theme
Environmental Context/Resources	• Comprehensive, interdisciplinary care and continuity • Accessing healthcare professionals and clinics
Beliefs about Consequences	• Medication characteristics • Treatment characteristics • Confidence and experience
Social Influences	• Stigma and discrimination • Relationships and supports

### Theme: Comprehensive, interdisciplinary care and continuity

Many barriers to accessing and prescribing buprenorphine raised across all perspectives related to the need for better continuity of comprehensive, interdisciplinary care for patients who need buprenorphine. This included the availability of and disjointed and unaligned nature of community mental and social services for people with living/lived expertise: *“What you have*,* essentially*,* is a delivery system which is entirely specialized….separate*,* siloed*,* specialized clinics*,* which is unheard of in any other medical or psychiatric condition*,*”* (Organizational leader, ID8).

While Rapid Access Addiction Medicine clinics were frequently lauded for supporting patients with follow-up addiction care for buprenorphine, participants emphasized the siloed nature of this care and the limited accessibility as they are not open every day. People with living/lived expertise, healthcare professionals, and organizational representatives stressed negative implications associated with other transitions between primary care and settings such as hospitals, correctional facilities, withdrawal management services, housing services, and residential treatment centres, including delayed and impeded buprenorphine treatment. Collaborations between clinics and pharmacies were seen as especially important to ensure that patients maintain their buprenorphine treatment. For example, organizational representatives and healthcare professionals expressed that when pharmacies are not co-located or near the clinic where the patient is initially prescribed buprenorphine, it creates cost and time barriers for both patients and prescribers:

*There’s a request that comes down to you to change the Suboxone dose and you send it to the pharmacy and then it’s like, the next time that the pharmacy sends up the prescriptions, the dose actually changes, so it can be, like up to a 10-day delay in the dose change, which I think, you know, people find frustrating* (Healthcare professional, ID13).

### Theme: Accessing healthcare professionals and clinics

Several environmental context/resources barriers to uptake and prescribing of buprenorphine were related to organizational limitations, such as recruiting and retaining qualified prescribers, infrastructure and capacity issues (e.g., space for private urine drug screening, nursing resources), and support from organizational leadership. The amount of time required for initiating, providing, and receiving buprenorphine treatment, and the complexities of scheduling and keeping appointments, were also frequently raised by all perspectives. People with living/lived expertise identified access to healthcare professionals, pharmacies, and support services for their buprenorphine treatment as a challenge, especially for those in rural locations or with limited transportation or mobility constraints. Some financial and funding concerns were noted across all perspectives, specifically limited resources for community-based services:

*But you fund us like crap comparatively. And you don’t have permanent positions. And we’re not funded like the other community health centers. …And you don’t have people knocking down the door to work here. Right? Because it’s hard work. And … you don’t have the resources to necessarily support that work* (Organizational leader, ID17).

However, public coverage of buprenorphine was more frequently noted as a facilitator to access for people with living/lived expertise and healthcare professionals.

### Theme: Medication characteristics

Desirable pharmacological characteristics were frequently mentioned facilitators for improving the use or support for buprenorphine from all perspectives. Evidence of buprenorphine’s effectiveness and safety profile were key drivers for its use among healthcare professionals and organizational representatives, including reduced risk of overdose and dosing errors. Much of buprenorphine’s effectiveness was attributed to the fact that it increases energy, improves mood, and increases mental clarity. All participants spoke about the positive effects of buprenorphine on functionality and quality of life, and reported that the benefits strongly outweighed any risks: “*They don’t have the ups and downs. They don’t have the cravings and that despair. It’s much more predictable*,* their life*,*”* (System, ID12).

However, the negative side effects of buprenorphine, especially bitter taste and the amount of time required to dissolve buprenorphine under the tongue, were noted to be a barrier to continuing to use buprenorphine among people with living/lived expertise. Improved mental clarity from buprenorphine was not always welcome for people whose prior opioid use had helped to block out memories of trauma. People with living/lived expertise discussed concerns about relapse when these memories resurfaced. Additionally, the effectiveness in blocking other opioids was seen as undesirable for patients interested in continuing their substance use and wanting to experience a high. A common barrier to initiating buprenorphine treatment for healthcare professionals, organizational leaders, and people with living/lived expertise though, was the requirement of being in partial withdrawal before starting the medication, and some healthcare professionals cited concerns about the risk of precipitated withdrawal as a barrier to prescribing.

*The challenge of having them in partial withdrawal when you start it is challenging because they are injecting probably seven, eight times a day - one, they don’t want to stop; and two, they’re not inclined to get into withdrawal. That’s what they’re trying to avoid* (Organizational leader, ID5)

### Theme: Treatment characteristics

More flexible buprenorphine treatment – related to dispensing requirements, physician visits, urine drug screening (UDS), and ability to access it in a primary care setting – was a commonly referenced facilitator for initiation and retention in treatment for organizational and, system leaders, as well as healthcare professionals, especially when contrasted with methadone treatment. Starting patients on buprenorphine in a primary care setting was another important facilitator for system and, organizational leaders, along with healthcare professionals since it decreased stigma and provided the opportunity to address other health issues that might otherwise be neglected. Several healthcare professionals noted advantages of initiating buprenorphine with patients in the emergency department when they were already in early withdrawal and a “captive audience.” People with living/lived expertise who were required to make daily pharmacy visits or undergo regular UDS named these as barriers to starting and staying in buprenorphine treatment and pursuing other goals including employment: “*It’s like - imagine - I mean*,* you know*,* like people who have diabetes*,* if they had to go to their pharmacist every day to get their insulin. I mean*,* that is a disincentive*,* like hugely inconvenient*,* right?” *(Person with living/lived expertise of opioid use, P9).

### Theme: Confidence and experience

Many healthcare professionals expressed apprehension about prescribing buprenorphine; some saw it as akin to anxiety with prescribing any new medication, while others had anxiety specific to opioid therapies and potential diversion. Nearly all healthcare professionals, system, and organizational representatives agreed that the confidence of prescribers would improve with experience though: *I don’t think that there’s a training barrier*,* I think it’s more having an opportunity to put the skills to practice*,*” (Organizational leader*,* ID16)*. For those who had not prescribed buprenorphine, were just starting, or were doing so infrequently, access to mentors with addictions expertise to consult was seen as important. When asked about intentions, healthcare professionals typically noted the importance of buprenorphine as one of the treatment options that should be available for patients. Healthcare professionals and people with living/lived expertise reiterated buprenorphine is just one “part of the puzzle” and there should be focus and resources to address all of the patients’ needs and priorities.

### Theme: Stigma and discrimination

In this TDF domain, barriers related to stigma and discrimination permeated our findings, with specific references to self, social, and structural stigma. People with living/lived expertise experienced discrimination by family and friends, the media, healthcare professionals, policies, and protocols, which impeded their access to buprenorphine. Some healthcare professionals described the unconscious biases and fears associated with people with substance use disorders amongst their colleagues, which impedes the prescribing of buprenorphine. Other healthcare professionals thought buprenorphine might decrease stigma for people in treatment because, unlike methadone, buprenorphine can be prescribed in non-specialized clinics in Ontario and is less well-known by the general public. Several organizational representatives remarked how stigma and discrimination towards addiction and people who use drugs has resulted in a lack of resources for this population and siloed care that does not address underlying causes of substance use disorders, including intergenerational trauma and the social determinants ofhealth:

*And we think of treating drug addiction as Suboxone and methadone, but we don’t talk about the physical and the mental health challenges, the cultural challenges, and then all of those social determinants that pushed them into this in the first place* (Organizational leader, ID5).

### Theme: Relationships and supports

Another commonly mentioned barrier to buprenorphine use in the social influence TDF domain was the quality of the patient and healthcare professional relationship. When this relationship was characterized by open communication and compassion, people with living/lived expertise perceived that their health needs and preferences were better met: “*The big thing here is focusing on relationships*,* right? So everything that we do is in that context of supporting and building that relationship that we have with our clients. I think it’s looking at the person holistically*,* right?”* (Healthcare professional, ID17). All perspectives discussed access to a supportive social support network as a facilitator to buprenorphine access and use, and the lack of one impeded patients’ ability to achieve their goals. Some people with living/lived expertise described that getting support can be complicated for patients beginning OAT who need to, paradoxically, disconnect from existing social supports who may not be helpful for their recovery.

## Discussion

Our study analyzed 28 qualitative interviews and applied the TDF to help understand barriers and facilitators to buprenorphine access and use experienced across people with living/lived expertise of opioid use, healthcare professionals, as well as organizational and system leaders. Across these levels, representatives reported barriers and facilitators that we categorized primarily in the TDF domains of environmental context/resources, beliefs about consequences, and social influences. Key cross-cutting themes included stigma/ discrimination, relationships and supports, medication and treatment characteristics, confidence and experience, as well as access to clinics and availability of comprehensive care.

Among varied perspectives on prescribing, people with living/lived expertise often described the medication characteristics, especially the negative side effects of buprenorphine as a concern. Meanwhile, organizational and system leaders, as well as healthcare professionals focussed more on the evidence of effectiveness and the flexible treatment characteristics as a facilitator for initiating and retaining people on buprenorphine. Healthcare professionals also expressed greater apprehension to prescribing due to their confidence and experience with buprenorphine. Among social factors, the concerns of people with living/lived expertise centred on the experiences of stigmatization and quality of relationships, while healthcare professionals noted the unconscious biases that impeded prescribing practices.

Barriers and facilitators in the knowledge and skills were rarely identified in this current study. In contrast, some previous literature from Vancouver has documented inadequate knowledge as a barrier to initiating buprenorphine for people with living/lived expertise [26] and healthcare professional training/skill development as a facilitator to implementation [15, 27], While this may be due to how we recruited participants, it is noteworthy as many strategies and investments often target knowledge gaps to improve buprenorphine implementation. Lanham et al. (2022) [26] surveyed U.S. clinicians to assess barriers and facilitators of obtaining an X-waiver and prescribing buprenorphine and found that one of the most frequently mentioned facilitators related to the need for mentorship and networks connecting experienced and knowledgeable clinicians to those contemplating or newer to prescribing. Our findings are consistent with this survey and others [28] that describe supports are needed beyond training to facilitate buprenorphine prescribing amongst healthcare professionals. Specifically, the need for ongoing mentorship [26] and access to substance use expertise to support the confidence and experience of healthcare professionals – for example with help and validation to apply knowledge and skills when and where they are needed in real-time – rather than focussing on general access to information.

We found the lack of interdisciplinary care and continuity and access to healthcare professionals and clinics were common barriers, which confirm the findings of other surveys and qualitative interviews [28, 29]. To improve access to buprenorphine more broadly, there is an opportunity to continue supporting the expansion and integration of historically siloed substance use services into primary care, emergency care, and other settings typically accessed by people with living/lived expertise of drug use. There is an existing body of evidence that supports collaborative care models with the integration of mental health and substance use and primary care services to be cost-effective and improve outcomes and access to care [29–33]. Increasing system capacity can address low access rates to primary care providers and team-based high-quality care among people treated for OUD [34] and can integrate substance use care with interventions to address social determinants of health, resulting in comprehensive, patient-centered care for people with living/lived expertise of drug use.

Our study also found facilitators for uptake included beliefs that buprenorphine was an effective and safe treatment that could offer more flexibility to reduce the burden of care and help enable people with living/lived expertise to function ‘normally’. Meanwhile, similar to previous studies assessing the patient experience of buprenorphine [8, 35, 36], some people with living/lived expertise faced barriers due to the lack of flexibility with treatment requirements. Recent guideline and regulatory changes to facilitate care for people with living/lived expertise during the COVID-19 pandemic (e.g., expanded virtual care, reduced clinic visit and UDS frequency, and increased access to unsupervised doses) [37] have been evaluated. Evaluations from the U.S. and Canada assessing the effects and experiences of COVID-19 changes on OAT access and prescribing demonstrate that reduced restrictions may improve retention and access for certain populations [38, 39], and not be associated with significant negative health outcomes [40, 41]. Further attention to the implementation of more flexible OAT guidelines is warranted as an opportunity to remove barriers and support the needs and priorities of people with living/lived expertise. With regard to the medication, people with living/lived expertise noted that the positive effects of using buprenorphine can be experienced negatively. For example, improved mental clarity can expose unresolved mental health concerns and trauma. To address this barrier, dedicated comprehensive, integrated care that involves mental health and peer support [42] and peer-led services are needed to support the health and social needs of people accessing buprenorphine treatment.

Where our study also found stigma and discrimination around substance use to be major barriers to buprenorphine across all levels, this has been identified in previous qualitative and review-level literature on the barriers and facilitators to buprenorphine or more broadly OAT [35, 43–46]. Participants noted stigma and discrimination towards addiction and people who use drugs influences the lack of resources, quality, and siloed nature of addiction care. Arguably, siloed models of OAT may draw resources away from more comprehensive approaches that address the critical upstream social determinants of health that are needed to improve the lives of people who use drugs. Efforts to enhance buprenorphine implementation must continue to prioritize addressing broader structural stigma towards people who use drugs and resource investments [47, 48], which may influence behaviours towards buprenorphine treatment at all levels. Such systemic anti-stigma efforts should be collaboratively designed with community members and organizations that provide services to people with living/lived expertise.

### Strengths and limitations

To our knowledge, this is the first qualitative study to apply a behaviour change framework to understand multi-level barriers and facilitators to address buprenorphine implementation. Analyzing barriers and facilitators within and across individual perspectives revealed important similarities and differences. Further, our theory-informed analysis is key to identifying barriers that may not be immediately associated with buprenorphine implementation and ensuring the development of evidence-based implementation strategies for addressing specific barriers. The next step of our project is to collaboratively map these barriers to strategies using behaviour change frameworks. The diverse expertise of our study team and Advisory Committee supported the interpretation and contextualization of the findings to current realities.

Study limitations include the evolving nature of the drug toxicity crisis in Ontario, which may limit the findings from our data that was collected between 2019 and early 2020. However, we believe that many of these barriers persist today, and have only been exacerbated by the growing contamination of fentanyl, other high-potency synthetic opioids, and adulterants in the unregulated drug supply [49, 50], COVID-19, and the housing affordability crisis, making the need for individualized buprenorphine treatment even more critical. While the use of the TDF helps us identify evidence-based opportunities to change behaviours, the use of additional complementary human rights and organizational frameworks would further our understanding to drive change. We acknowledgement that the depth within each area was limited by taking a broad system perspective and also restricted by time and resources for this one-year project. Of note, the small sample and representation of Northern, Eastern, and Western Ontario limit the external generalizability of our results and should be considered when considering the rest of Ontario, other Canadian provinces, countries, and population groups. While the small sample of participants across the four levels limited the ability to achieve saturation for each, we were able to identify broad themes, similarities, and differences across perspectives. Further work is required to explore barriers and facilitators through an equity lens including race, ethnicity, gender, sex, and other characteristics associated with discrimination. Lastly, we purposively identified participants based on our networks who may have been more knowledgeable about and involved in buprenorphine use and prescribing and not representative of the general population within all levels; however, we used a matrix of characteristics to plan the recruitment of diverse participants in Ontario.

## Conclusion

Barriers to accessing and using evidence-based buprenorphine treatment are experienced at multiple levels of the healthcare system during the drug toxicity crisis in Ontario. Our study found common themes of barriers and facilitators that cut across the perspectives of people with living/lived expertise of opioid use, healthcare professionals, as well as organizational and system leaders. These include: environmental context/resources, beliefs about consequences, and social influences. Our results support the need for mentorship models of support for healthcare professionals, flexibility in buprenorphine treatment requirements, better recognition of the mental health and social determinants of health in buprenorphine treatment, and developing comprehensive, integrated care systems for people who use drugs.

## Supplementary Information

Below is the link to the electronic supplementary material.

Supplementary Material 1

Supplementary Material 2

Supplementary Material 3

Supplementary Material 4

## Data Availability

Data generated in the study are not publicly available to protect the privacy and anonymity of participants.
