# Supplementary material for: Multi-level barriers and facilitators to buprenorphine use in Ontario, Canada: a qualitative study using the theoretical domains framework
Source: Addict Sci Clin Pract. 2025 Oct 21;20:83. doi: 10.1186/s13722-025-00610-w (PMC12538829; doi:10.1186/s13722-025-00610-w)
Supplement: Supplementary file 1 — Supplementary Material 1 [file 13722_2025_610_MOESM1_ESM.docx]

**Additional File 1.**

**Table 1. Domains of the Theoretical Domains Framework**

| **TDF Domain** | **Description** | **Applied to behaviours related to buprenorphine** |
| --- | --- | --- |
| **Environmental context/resources** | Any circumstance of a person’s situation or environment that discourages or encourages the development of skills and abilities, independence, social competence, and adaptive behaviour | Circumstances in the environment that discourage or encourage accessing, prescribing, or enabling use of buprenorphine |
| **Beliefs about consequences** | Acceptance of the truth, reality, or validity about outcomes of a behaviour in a given situation | Thoughts about outcomes of using, accessing, or prescribing buprenorphine |
| **Social influences** | Those interpersonal processes that can cause individuals to change their thoughts, feelings, or behaviours | Interpersonal processes that can cause individuals to change thoughts and behaviors related to accessing, prescribing, or enabling use of buprenorphine |
| **Professional/social role and identity** | A coherent set of behaviours and displayed personal qualities of an individual in a social or work setting | A set of clear behavior and personal qualities of an individual in a social or work setting related to the use of buprenorphine |
| **Reinforcements** | Increasing the probability of a response by arranging a dependent relationship, or contingency, between the response and a given stimulus | Process of encouraging or establishing a belief or pattern of behavior related to buprenorphine by encouragement or reward, and also by punishment or disincentives |
| **Emotion** | A complex reaction pattern, involving experiential, behavioural, and physiological elements, by which the individual attempts to deal with a personally significant matter or event | A complex reaction pattern related to accessing, prescribing, or enabling use of buprenorphine |
| **Knowledge** | Awareness of the existence of something | Awareness of the existence or use of buprenorphine |
| **Behavioral regulation** | Anything aimed at managing or changing objectively observed or measured actions | Managing or changing actions related to accessing, prescribing, or enabling use of buprenorphine |
| **Beliefs about capabilities** | Acceptance of the truth, reality, or validity about an ability, talent, or facility that a person can put to constructive use | Thoughts about the ability to access, prescribe or enable buprenorphine use that a person can put to constructive use |
| **Optimism** | The confidence that things will happen for the best or that desired goals will be attained | Confidence that things will happen for the best or that desired goals related to buprenorphine will be attained |
| **Intentions** | A conscious decision to perform a behaviour or a resolve to act in a certain way | Conscious decision to access, prescribe, or enable buprenorphine use |
| **Goals** | Mental representations of outcomes or end states that an individual wants to achieve | An aim or desired result an individual wants to achieve in terms of accessing, prescribing, or enabling use of buprenorphine |
| **Skills** | An ability or proficiency acquired through practice | Ability to use, prescribe, or implement buprenorphine |
| **Memory, attention and decision processes** | The ability to retain information, focus selectively on aspects of the environment and choose between two or more alternatives | Ability to retain information, focus, and choose between two or more alternatives, or decisions during ongoing treatment (e.g., dose) |
