# Supplementary material for: Multi-level barriers and facilitators to buprenorphine use in Ontario, Canada: a qualitative study using the theoretical domains framework
Source: Addict Sci Clin Pract. 2025 Oct 21;20:83. doi: 10.1186/s13722-025-00610-w (PMC12538829; doi:10.1186/s13722-025-00610-w)
Supplement: Supplementary file 3 — Supplementary Material 3 [file 13722_2025_610_MOESM3_ESM.docx]

**Additional File 3.**

**Variations by perspective**

About half of the codes for people with living/lived expertise of opioid use fell in the domain of beliefs about consequences (51%), compared with fewer than one-fifth of the other perspectives. The reverse pattern was true for environmental context/resources; this domain garnered about one-fifth of the codes for people with living/lived expertise of opioid use, compared with about two-fifths for the other perspectives. The third most common domain was social influences; about one-fifth of people with living/lived expertise of opioid use and healthcare professional codes, compared with one-tenth of organizational and system-level codes, were in this domain. Together, nearly nine out of ten of the barriers and facilitators identified by people with living/lived expertise of opioid use fell into these three domains, and approximately two-thirds of those identified at healthcare professional, organization, and system perspectives.

**Table 1. Coding frequencies by TDF domain and perspective**

| **TDF Domain** | **Number of codes**  **(N=1,194)**  **n (%)** | **People with living/lived expertise of opioid use**  **(n=344)** | **Healthcare professionals**  **(n=307)** | **Organizational**  **(n=286)** | **Systems**  **(n=257)** |
| --- | --- | --- | --- | --- | --- |
| Knowledge | 35  (3%) | 2 | 11 | 10 | 12 |
| Skills | 11  (1%) | 0 | 2 | 6 | 13 |
| Memory, Attention and Decision Processes | 11  (1%) | 3 | 1 | 2 | 5 |
| Behavioral Regulation | 34  (3%) | 0 | 12 | 12 | 10 |
| Environmental Context/Resources | 408  (34%) | 71 | 112 | 121 | 104 |
| Social Influences | 129  (11%) | 52 | 33 | 24 | 20 |
| Intentions | 29  (2%) | 12 | 7 | 5 | 5 |
| Beliefs about Consequences | 317  (27%) | 176 | 59 | 41 | 41 |
| Goals | 26  (2%) | 8 | 7 | 3 | 8 |
| Professional/Social Role and Identity | 43  (4%) | 1 | 13 | 19 | 10 |
| Beliefs about Capabilities | 21  (2%) | 1 | 10 | 6 | 4 |
| Optimism | 25  (2%) | 4 | 7 | 6 | 8 |
| Reinforcements | 52  (4%) | 5 | 13 | 17 | 17 |
| Emotion | 53  (4%) | 9 | 20 | 14 | 10 |
