# Supplementary material for: Multi-level barriers and facilitators to buprenorphine use in Ontario, Canada: a qualitative study using the theoretical domains framework
Source: Addict Sci Clin Pract. 2025 Oct 21;20:83. doi: 10.1186/s13722-025-00610-w (PMC12538829; doi:10.1186/s13722-025-00610-w)
Supplement: Supplementary file 4 — Supplementary Material 4 [file 13722_2025_610_MOESM4_ESM.docx]

**Additional File 4.**

**Table 1. Additional Supporting Quotes**

| **Theme** | **Perspective** | **Excerpts** |
| --- | --- | --- |
| **ENVIRONMENTAL CONTEXT/RESOURCES** | | |
| ***Comprehensive, interdisciplinary care and continuity*** | People with living/lived expertise of opioid use | - [Barrier] *There has to be a clean transfer between, like, if [patients] have to go to jail, their prescription needs to be moved automatically or, you know, reliably … because their lives are at stake.* (P10) |
|  | Organization | - [Barrier] *So, transportation, ID [identification], ability to get into a clinic or ability to get their narcotics certainly are all of those things are things I’m thinking of when I’m starting someone. I don’t want to get them on Suboxone [buprenorphine] successfully in hospital and not be able to transition them to the community. I want to make sure it’s a safe and effective and successful transition.* (ID7) |
|  | System | - [Barrier] *how do you get the mental health counsellors, how do you get the nursing support … it’s not a walk-in clinic where you’re seeing, you know, urinary tract infections, you do need some mental health support to really make it effective, I would think. I think there is the availability of those resources in the system, the problem is that they’re not aligned and hopefully, through things like Ontario Health Teams, they will become aligned.* (ID14) |
| ***Accessing healthcare professionals and clinics*** | People with living/lived expertise of opioid use | - [Barrier] *He couldn’t afford a car, we had to lend him our car, and it was like, over half-hour drive into town to where he had to go. The challenge was having his pee tests and the frequency of those and having to drive further to get that done. … like I think it was twice a week. And it caused quite a lot of problems, because when he went into town, then he would socialize with his friends who are using as well, and also, he would be stealing. It was very challenging. The amount of appointments and the hours and the - especially rural, when you live rurally, it’s very difficult to get help.* (P10) - [Facilitator] *Well if I had to pay for it, I wouldn't be able to afford it. Not at all.* (P11) |
|  | Healthcare professional | - [Facilitator] *I think like having a local expert can definitely enforce good practice. It was nice to have {physician’s name} who has experience with Suboxone, to consult to, bounce ideas off of. (ID15)* - [Facilitator] *The fact that it’s covered now by ODB [Ontario Drug Benefit] … there are so many patients that benefitted when that came through* (ID1) |
|  | Organization | - [Barrier] *I think in the community addiction medicine clinics, many of them largely work off of walk-ins or sort of non-scheduled appointments. And so I think for us it’s easier to provide Suboxone care to patients who can keep appointments. And less easy for us to provide it to patients who can’t keep appointments. (ID16)* - [Barrier] *The way that we are funded is systemically racist…. Why would you expect people to provide quality care to people who…probably need a lot more care than your general family health team practice, even your general community health centre practice right? Because people in a community health centre are not facing the same systemic barriers as Indigenous people. (ID17)* |
|  | System | - [Barrier] *It’s very time consuming to be available to those patients when they are having the transition and having withdrawal.* (ID12) |
| **BELIEFS ABOUT CONSEQUENCES** | | |
| ***Medication characteristics*** | People with living/lived expertise of opioid use | - [Facilitator] *I think Suboxone [buprenorphine] is a miracle drug.* (P9) - [Facilitator] *Now I'm on this and I feel so much better. It's helped me tremendously. I'm glad. I'd be dead if I didn't have this right now. I'd be dead. I wouldn't be sitting here. …Yeah. It saved my life it did.* (P11) - [Facilitator] *[It’s] a lot easier to just be you.* (P2) |
|  | Healthcare professional | - [Facilitator] *I feel like it was a really good drug for safety. You know, especially if we do have people that decide to do some IV drug use and that sort of thing. Just the naloxone component of it makes it that much more safe. Also, too, is I have people that aren't necessarily compliant with medications. If they miss a dose, it's not the end of the world …. So, I don't have to worry about their tolerance being adjusted and accidentally overdosing themselves if they miss a couple doses and then go back to the same dose in the same way, let's say, a methadone or any of the other medication, opioids, might be. So, I find it's just the safety profile of it is a big advantage over the other opioids.* (ID10) |
|  | Organization | - [Facilitator] *They’re able to maintain the quality of life that they desire for themselves. (ID9)* |
| ***Treatment characteristics*** | People with living/lived expertise of opioid use | - [Barrier] *I also had a parent just last week, express, again, her son really wants to get a job, but he just feels like it’s almost impossible because of the daily dispensing of Suboxone, you know? I think funding of the other - the other formats of it, or whatever that’s the right word, it’s not format, but you know, the Sublocade, the Probuphine, the things that make it easier to, you know, to use. … (P9)* |
|  | Healthcare professional | - [Facilitator] *I think that there is a huge advantage in having the family physician doing it, because you’re seeing them probably more frequently… You feel comfortable hopefully with your family doctor, you have a relationship with your family doctor that can be leveraged. (ID15)* |
|  | Organization | - [Facilitator] *Managing things that are chronic medical issues that would may otherwise be neglected. Things like diabetes…HIV, hepatitis C, COPD, immunizations. A lot of these patients would not necessarily come in just to deal with these issues, but [may engage] because they’re with you in the office; (ID16)* - [Facilitator] *It’s less rigid, less structured... the dispensing issues are not as prominent, dosing more flexible. (ID5)* |
|  | System | - [Facilitator] *I think there’s definitely a better convenience factor for patients, as opposed to patients who are tied to having to go to a methadone clinic and having their urine tested all the time. (ID14)* |
| ***Confidence and experience*** | People with living/lived expertise of opioid use | - [Facilitator] *I think that it’s one part of the puzzle for helping people with substance use disorder, but I think it’s only part of the puzzle and there has to be other resources available to help people. (P10)* |
|  | Healthcare professional | - [Barrier] *With any sort of new medication if you've never done it you're apprehensive I think. (ID17)* - [Barrier] *There’s a lot of fear around, it’s not just buprenorphine, it’s a fear of opioid therapies in general, and nurse practitioners, we only got the ability to prescribe controlled substances a few years ago now, I’d say. So, it’s really new to our field, and there is a fear about it. (ID10)* - [Barrier] *You feel very vulnerable from your licence point-of-view, that you don’t really know if the substance is being diverted; (ID13)* |
|  | System | - [Facilitator] i*f the system can support people who are interested in becoming buprenorphine naloxone prescribers, they need not only the knowledge - the knowledge is good, everybody needs to know about it - but those when they start, they need this coaching and mentoring, a few hours here and there. (ID12)* |
| **SOCIAL INFLUENCES** | | |
| ***Stigma and discrimination*** | People with living/lived expertise of opioid use | - [Barrier] *Most people who are struggling with opioid use disorder, a lot of them have self-esteem issues and just, honestly, how they’re treated makes it worse, you know? It’s the opposite of therapeutic. (P9)* |
|  | Healthcare professional | - [Barrier] *The whole stigma of caring for “that population”, right, they see it as there’s this otherness to it, they don’t see that this is us. (ID3)* |
|  | Organization | - [Barrier] *But this whole idea that many emerg [Emergency Department] docs have, is yeah, I don't like these patients, I don't want them here, and besides, they should go to the methadone clinic. That's got to change. I mean, I know why it's possible. It's because these people have no political voice and…the concern about them is not there the way it would be for some condition that affects middle class, but you know, that's the way it is, I guess. So, it really [will], I think, be considered a scandal in decades to come, about our poor and completely inadequate response to the opiate crises. (ID8)* - [Barrier] *They’ve got to be housed. They’ve got to get back to school. They’ve got to get a job. They’ve got to earn an income, you know? All those things that ... and get back with their family and supports. All those things are enormously challenging for them.(ID5)* |
|  | System | - [Barrier] *If there are pain patients and it's hard for you to taper, then buprenorphine naloxone may be a good option. But then… [other] patients were saying if you accept this drug it will go on your medical records, your permanent health records, this diagnosis that you are an opioid addict will never leave you. (I12)* |
| ***Relationships and supports*** | People with living/lived expertise of opioid use | - [Facilitator] *I’ve got a lot of support here, and thank God, if I didn’t have the support here, I don’t think I would have pulled through. (P11)* |
|  | Organization | - [Facilitator] *They have a very longstanding history of using drugs to address issues in their lives that would be setbacks, that you or I might, you know ... have a difficult day and complain to our spouses and ... but we’ve got supports around us that will help us through those difficult days. They, historically, have when something’s gone wrong in their lives, a relationship, violence, abuse, whatever it might be, it triggers so much and they just go immediately back to their safe zone, which for them is going back to drugs. (ID5)* |
|  | System | - [Barrier] *And people should be allowed to choose their own care and they should have access to what that type of care is (ID4)* - [Facilitator] *I have patients who are established and working and are at risk of losing a lot. And they're usually able to pay, and they tend to do better, because, you know, they’ve got great social supports. ID1)* |
